# Distinct genome stabilization procedures lead to phenotypic variability in newly generated interspecific yeast hybrids

**DOI:** 10.3389/fmicb.2025.1472832

**Published:** 2025-01-29

**Authors:** Pablo Murath, Stephanie Hoffmann, Beatriz Herrera-Malaver, Luis Bustamante, Kevin Verstrepen, Jan Steensels

**Affiliations:** ^1^Departmento de Análisis Instrumental, Facultad de Farmacia, Universidad de Concepción, Concepción, Chile; ^2^Departamento de Ciencia y Tecnología de los Alimentos, Facultad de Farmacia, Universidad de Concepción, Concepción, Chile; ^3^Leuven Institute for Beer Research, Katholieke Universiteit Leuven, Bio-Incubator, Leuven, Belgium; ^4^Laboratory for Systems Biology, VIB Centre for Microbiology, Bio-Incubator, Leuven, Belgium; ^5^Laboratory for Genetics and Genomics, Centre of Microbial and Plant Genetics (CMPG), Katholieke Universiteit Leuven, Leuven, Belgium

**Keywords:** Lager yeast, interspecific hybrids, genome stabilization, flavor production, brewing

## Abstract

Yeast cells sometimes engage in interspecific hybridization, i.e., crosses between different species. These interspecific yeast hybrids combine phenotypes of the two parental species and can therefore allow fast adaptation to new niches. This is perhaps most evident in beer yeasts, where a cross between *Saccharomyces cerevisiae* and *Saccharomyces eubayanus* led to the emergence of the lager yeast *Saccharomyces pastorianus*, which combines the fermentation capacity of *S. cerevisiae* with the cold tolerance of *S. eubayanus*, making the hybrid suitable for the typical cool lager beer fermentation conditions. Interestingly, however, merging two different genomes into one cell causes genomic instability and rearrangements, ultimately leading to a reorganized but more stable hybrid genome. Here, we investigate how different parameters influence this genome stabilization trajectory and ultimately can lead to variants with different industrial phenotypes. We generated seven *de novo* interspecific hybrids between two *S. eubayanus* strains and an ale *S. cerevisiae* strain, subsequently exposing them to three different genome stabilization procedures. Next, we analyzed the fermentation characteristics and metabolite production of selected stabilized hybrids. Our results reveal how variation in the genome stabilization procedure leads to phenotypic variability and can generate additional diversity after the initial hybridization process. Moreover, several stabilized hybrids showed phenotypes that are interesting for industrial applications.

## 1 Introduction

Interspecific hybridization has been identified as an important driver of yeast evolution. Crosses between different yeast species lead to hybrids that combine phenotypes of both parental species, which may allow them to thrive in new niches (Steensels et al., 2021). Whereas the resulting hybrids are typically sexually infertile, they can reproduce asexually through mitotic budding.

While interspecific hybridization occurred across the entire yeast phylogeny, many examples have been identified within the *Saccharomyces* species complex (Gallone et al., 2019; Morales and Dujon, 2012; Steensels et al., 2021). Despite the high sequence divergence between the species within this genus (up to 20%) (Kellis et al., 2003), prezygotic barriers are generally weak, and species can successfully cross-breed. Given that interspecific hybridization can act as an evolutionary fast-track towards adaptation to new environmental niches, it is perhaps not surprising that interspecific *Saccharomyces* hybrids are often encountered in novel niches created by humans. For example, interspecific hybrids were identified in Belgian traditional ales (Gallone et al., 2019; Peris et al., 2012, 2018), wine (Christine et al., 2007; González et al., 2006, 2007; Lopes et al., 2010; Masneuf et al., 1998), and bread (Marcet-Houben and Gabaldón, 2015). The most famous and industrially most relevant example is *S. cerevisiae x S. eubayanus* (also referred to as *S. pastorianus*), which is used to produce lager-type beers (Bing et al., 2014; Eizaguirre et al., 2018; Libkind et al., 2011; Walther et al., 2014).

Beer is a fermented beverage that, according to archeological evidence, has been brewed since the beginning of human civilization by Sumerians in the last half of the fourth millennium BC (Michel et al., 1992), possibly even dating back as far as 13,000 years ago (Liu et al., 2018). Today, beer is the most-produced fermented beverage, with an annual production of 189 billion liters in 2022 (Statista, 2024), with lager beer representing around 80–90% of that total beer volume (Cimini and Moresi, 2021). Lager beer originated in 16th century Bavaria, as a result of the Bavarian Beer Purity Law (Bayerisches Reinheitsgebot) (Hutzler et al., 2023). The primary objective of this law was to limit bacterial contaminations during beer production by establishing rules regarding ingredients and restricting brewing to the colder winter months. Hence, lager beers are typically fermented at lower temperatures compared to the older, more traditional ale beers, which are fermented at higher temperatures by *S. cerevisiae* yeast cells. The adaptation of cooler fermentation temperatures instructed by the Reinheitsgebot likely led to the emergence of *S. pastorianus*, when a cell of *S. cerevisiae* hybridized with a wild *S. eubayanus* cell. The resulting interspecific hybrid combined the ability of S. *cerevisiae* to thrive in the sugar-rich beer medium with the cold-tolerance of *S. eubayanus*, enabling it to outcompete its parents (Gibson and Liti, 2015; Gibson et al., 2013; Mertens et al., 2015). Interestingly, molecular clock analyses indicated this new species emerged not long after lager brewing was established (Gallone et al., 2019), highlighting how quickly interspecific hybrids can colonize new niches.

Genetic analyses have revealed that lager strains can be divided in two distinct lineages, which are called Saaz (Type 1) and Frohberg (Type 2) (Dunn and Sherlock, 2008; Liti et al., 2005; Monerawela et al., 2015). Although it is still unclear whether these two lineages originate from two distinct interspecific hybridization events, it is well known that the two types show distinct characteristics. Saaz strains are generally allotriploids with proportionally more DNA from the parental *S. eubayanus* (Gallone et al., 2019; Walther et al., 2014), while Frohberg strains are allotetraploids with predominantly DNA from the *S. cerevisiae* parent (Gallone et al., 2019; Nakao et al., 2009; Okuno et al., 2015). Despite the existence of these two lineages, genetic and phenotypic variability among lager yeasts is still very low, especially compared to that of ale yeasts (Gallone et al., 2019).

The discovery of the first pure *S. eubayanus* strain in 2011 (Libkind et al., 2011), provided the opportunity to mimic the original *S. pastorianus* hybridization event in the lab, resulting in new *S. cerevisiae x S. eubayanus* hybrids (Hebly et al., 2015; Krogerus et al., 2015, 2018; Mertens et al., 2015). The resulting interspecific hybrids can show a higher fermentation capacity (i.e., faster fermentation and higher attenuation) than both parental strains in lager beer fermentations. Moreover, some of these novel hybrids produced flavor profiles differing drastically from those of currently available Frohberg or Saaz strains (Mertens et al., 2015).

In addition to the phenotypic variability generated by the hybridization process itself, several studies suggest that the resulting hybrids have unstable genomes, and that subsequent postzygotic evolution in selective conditions further influences the genetic and phenotypic properties of the hybrids. Indeed, genomic redundancy after the hybridization event can cause chromosomal losses, duplications and recombination (Gorter De Vries et al., 2019; Krogerus et al., 2016; Lancaster et al., 2019; Piotrowski et al., 2012; Steensels et al., 2021; van den Broek et al., 2015), while the high level of heterozygosity introduced by hybridization further provides substrate for evolution to act on, for example by introducing regions of loss-of-heterozygosity, causing alleles of one of the parental species to become homozygous (D’Angiolo et al., 2020; Mozzachiodi et al., 2021; Smukowski Heil et al., 2017, 2019). Therefore, exposing newly formed interspecific hybrids to different stressors can result in variants with different phenotypic properties (Krogerus et al., 2018; Kunicka-Styczyńska and Rajkowska, 2011). In Saaz and Frohberg lager yeast, continuous evolution and selection during hundreds of years of beer fermentation cycles likely led to stabilized variants with properties differing from the initial hybrid cell. One particularly interesting example is the inability of lager yeast to convert ferulic acid to 4-vinylguaiacol (4-VG). 4-VG production is caused by the combined activity of two genes, *PAD1* and *FDC1*, and if at least one of these genes is absent or inactivated, 4-VG production does not occur (Gallone et al., 2019; Krogerus et al., 2017b; Mukai et al., 2010). Interestingly, many domesticated yeasts, including Frohberg and Saaz lager strains, lost or acquired mutations in these genes, rendering them 4-VG negative (Gallone et al., 2018, 2019). In contrast, most wild yeast, including all *S. eubayanus* strains isolated so far, still have active variants of these genes (Gallone et al., 2019; Steensels and Verstrepen, 2014). Another example of postzygotic evolution in lager yeasts is the emergence of specific transporters for maltotriose, improving their fermentation efficiency in wort (Brickwedde et al., 2017). Interestingly, similar transporters were obtained after evolving *S. eubayanus* in medium with maltotriose as sole carbon source (Baker and Hittinger, 2019; Brouwers et al., 2019), showing the potential of mimicking the historical hybridization and postzygotic genome stabilization event in a lab environment to generate new strains for lager brewing.

In this study, we developed seven novel interspecific hybrids (H1-H7) and subjected them to genome stabilization in three distinct stress conditions (Supplementary Figure S1). Specifically, beer wort of 12°P at 10°C (low temperature; LT), beer wort of 12°P at 10°C supplemented with 8% v/v ethanol (low temperature + high ethanol; LT+Eth) and beer wort of 12°P at 30°C (high temperature; HT) were used. For each of the hybrids, four parallel lineages were evolved in each condition. A selected subset of the evolved lineages was subsequently evaluated in lager beer fermentation conditions, and their metabolite production was determined. It became apparent that genome stabilization indeed affects hybrid performance, albeit the observed differences are modest. Interestingly, two individual stabilized isolates (H5_HT_L3i and H5_HT_L4i) consistently showed trends of higher production of several higher alcohols (isobutanol, isoamyl alcohol) and esters (ethyl acetate, isobutyl acetate, isoamyl acetate and 2-phenylethyl acetate), as well as total ethanol production. In addition, H5_HT_L4i showed lower production of 4-VG compared to other stabilized isolates of H5, and could therefore be an interesting strain to explore industrially.

## 2 Materials and methods

### 2.1 Yeast strains

Two strains of domesticated *S. cerevisiae* were selected based on their production of desirable aromatic compounds, sporulation capacity, and spore viability. Both selected strains of *S. cerevisiae*, BE031 and BE011, belongs to different industrial yeast sublineage, classified as Beer 1 and Beer 2, previously described by Gallone and coworkers (Gallone et al., 2016). Also, two newly isolated wild *S. eubayanus* strains from Chile (36°52’S–71°22’O) were used as parental strains. Two different *S. pastorianus* strains, corresponding to each type of lager yeasts - Saaz (CBS1513) and Frohberg (W34/70)—were included in the different experiments as reference strains.

### 2.2 Sporulation and tetrad dissection of possible parental strains

Sporulation of selected parental strains was induced on acetate medium (1% [wt vol^–1^] potassium acetate, 0.05% [wt vol^–1^] amino acid mix, and 2% [wt⋅vol^–1^] agar) after 5–10 days at 25°C. Subsequently, sporulation capacity was assessed using a light microscope (magnification of X40). The ascus wall was digested in 8 mL 0.1 mg mL^–1^ zymolyase (100 T, Amsbio) incubated overnight (35°C, 80 rpm). The suspension was centrifuged and resuspended in Triton X-100 1,5% [wt vol^–1^]. Tetrads were sonicated (Branson Digital Sonifier) in three cycles with amplitude 20% for 30 s, with a pause of 30 s between each cycle. Spores were stored at -80°C in glycerol-yeast extract-peptone-dextrose (GYPD) medium (2% [wt vol^–1^] Bacto peptone, 1% [wt vol^–1^] yeast extract, 2% [wt vol^–1^] dextrose, and 25% [wt vol^–1^] glycerol).

### 2.3 Hybrid generation through spore-to-spore mating

Hybridization was induced by placing single spores from both parental strains together with a micromanipulator (MSM; Singer Instruments) on YPD agar (2% [wt vol^–1^] Bacto peptone, 1% [wt vol^–1^] yeast extract, 2% [wt vol^–1^] glucose, and 1.5% [wt vol^–1^] agar), followed by visual inspection of zygote formation after 6–8 h of incubation at room temperature. Candidate interspecific hybrids were purified by streaking on synthetic 12 degrees Plato (°P) malt agar medium (12% [wt vol^–1^] synthetic malt extract [8EBC; Brouwland, Belgium] and 1.5% [wt vol^–1^] agar).

### 2.4 Interspecific hybrids confirmation

The confirmation of the newly generated interspecific hybrids was done by species-specific multiplex PCR analysis. Two primer pairs were used for the species-specific multiplex PCR, each targeting a specific part of one of the parental species genome (Muir et al., 2011; Pengelly and Wheals, 2013). Primers Scer F2 (5′-GCG CTT TAC ATT CAG ATC CCG AG-3′) and Scer R2 (5′-TAA GTT GGT TGT CAG CAA GAT TG-3′) amplify a 150-bp amplicon of the *S. cerevisiae* genome. Primers Seub F3 (5′-GTC CCT GTA CCA ATT TAA TAT TGC GC-3′) and Seub R2 (5′-TTT CAC ATC TCT TAG TCT TTT CCA GAC G-3′) generate a 228-bp *S. eubayanus*-specific amplicon. The PCR conditions were as follows: 3 min at 95°C, 30 cycles of 30 s at 95°C, 30 s at 58°C, and 30 s of 72°C, followed by a final cycle of 5 min at 72°C and subsequent cooling to room temperature (RT). Candidate hybrids showing two bands were considered to be interspecific hybrids. Single colonies of each interspecific hybrid were propagated in YPD growth medium (2% [wt vol^–1^] Bacto peptone, 1% [wt vol^–1^] yeast extract and 2% [wt vol^–1^] dextrose) and then stored at -80°C in GYPD medium.

### 2.5 Exposure of interspecific hybrids to different environmental conditions

The interspecific hybrids obtained and the parental strains were subjected to a genetic stabilization protocol. Four parallel lineages of each hybrid were individually inoculated into 100 μL of three different media conditions: 12°P wort medium at 10°C (LT), 12°P wort medium at 10°C with ethanol 8% (LT+Eth), 12°P wort medium at 30°C (HT) in a 96-well plate and incubated at 600 rpm for 1–3 days. After incubation, cultures were diluted 10 times in 90 μL of each fresh medium described above. This incubation/dilution procedure was repeated 6 times until reach around 20–25 yeast generations, an amount reported to be sufficient to achieve genomic stabilization for interspecific hybrids (Pérez-Través et al., 2015). Thereafter, all the evolved cultures were stored at -80°C. Cultures were plated on synthetic 12 degrees Plato (°P) malt agar medium (12% [wt vol^–1^] synthetic malt extract [8EBC; Brouwland, Belgium] and 1.5% [wt vol^–1^] agar, and for each culture, a single colony was isolated for phenotypic experiments.

### 2.6 Absorbance-based 4-VG measurement

The ability of the yeasts to produce 4-VG was tested via the absorbance-based detection method, described by Mertens et al. (2017). Yeasts were inoculated in 150 μL liquid YPD 2% growth medium, supplemented with 100 mg L^–1^ ferulic acid in a 96 well plate. In each plate, a 4-VG^–^ (W34/70) and a 4-VG^+^ (*S. mikatae* NCYC2888) control were included. Each stabilized lineage and control strain was tested in biological and technical replicates. 96-well plates were sealed with an aluminum cover foil and incubated for 5 days at 30°C, 200 rpm. After centrifugation (3 min, 3000 rpm), 100 μL of the supernatant was transferred to a new 96 well plate and remaining concentration of ferulic acid was measured at a wavelength of 325 nm (Tecan Infinite 200 PRO, Switzerland).

### 2.7 Laboratory-scale lager fermentations

Yeasts were propagated by inoculating in 5 mL YPD 2%, incubated at room temperature in a rotating wheel. After 16 h of incubation, 1 mL of the culture was transferred to 50 mL 4% yeast extract peptone-maltose (YPM; 2% [wt vol^–1^] Bacto peptone, 1%[wt vol^–1^] yeast extract, and 4% [wt vol^–1^] maltose) in a 250 mL Erlenmeyer flask and incubated at 20°C and 200 rpm for 72 h. Cell concentration was measured (BioRad, TC20 automated cell counter, USA), and 150 mL of an 12°P wort (12% [wt vol^–1^] Light spray malt extract (Brewferm, Belgium), supplemented with 0.005 mg L^–1^ Zn^2+^ (autoclaved for 10 minutes at 110°C) was inoculated with 10^7^ cells mL^–1^. The bottles were equipped with a water lock and stirring bar after which they were incubated at 14°C, agitated at 150 rpm. After 8 days, the fermentations were stopped and samples for chromatographic analysis and ethanol measurements were taken. The weight loss of the samples was measured daily. Samples for alcohol by volume analysis were analyzed by Alcolyzer beer ME (Anton Paar GmbH) according to the manufacturer recommendations. Sugar concentrations were determined by Dionex Liquid Chromatography (Thermo Fisher Scientific, United States), which was calibrated for maltotriose, maltose, sucrose and glucose using raffinose as the internal standard.

### 2.8 Higher alcohols, esters, and 4-VG measurement

Headspace gas chromatography coupled with flame ionization detection (HS-GC-FID) was used for the quantification of yeast aroma production (Agilent Technologies, United States), which was calibrated for the quantification of 13 important aroma compounds, including higher alcohols (propanol, butanol, isobutanol, isoamyl alcohol and 2-phenylethanol), esters (ethyl acetate, isobutyl acetate, isoamyl acetate, 2-phenylethyl acetate, ethyl butyrate, ethyl hexanoate and ethyl octanoate), and 4-vinyl guaiacol. The GC was equipped with a headspace autosampler (PAL system; CTC Analytics, Zwingen, Switzerland) and contained a DBWAXeter column (length, 30 m; internal diameter, 0.25 mm; layer thickness, 0.5 μm [Agilent Technologies, Santa Clara, CA]), and N2 was used as the carrier gas. Samples were heated for 25 min at 70°C in the autosampler. The injector block and FID temperatures were both kept constant at 250°C. 5 mL of sample was collected in 15 mL glass tubes containing 1.75 g of sodium chloride each. These tubes were immediately closed and cooled to minimize evaporation of volatile compounds. The oven temperature was held at 50°C for 5 min, after which it was increased to 80°C at 4°C min^–1^. Next, the temperature was increased to 200°C at 5°C min^–1^ and held at 200°C for 3 min. Results were analyzed with Agilent Chemstation software (Agilent, Santa Clara, CA).

### 2.9 Data analysis and data visualization

All the results were expressed as arithmetic means and standard error of the mean. Statistical analyses of the overall conversion of ferulic acid and statistical analyses between the parallel lineages and the overall production of higher alcohols, esters and 4-VG by the selected interspecific hybrids evolved in different conditions were assessed by Kruskal-Wallis one-way analysis of variance test in combination with Dunn‘s multiple comparison tests (α = 0.05) using GraphPad Prism version 10.0 (GraphPad Software). Furthermore, the average production of the different higher alcohols and esters was assessed by multiple Mann-Whitney tests (α = 0.05) using GraphPad Prism version 10.0 (GraphPad Software). Heatmap analysis and visualization was processed by freely accessible software MetaboAnalyst 5.0.^1^ Principal component analysis (PCA) analysis and visualization were performed in R using the FactoMineR package version 2.11, the factoextra package version 1.0.7 and ggplot2 package version 3.5.1.

## 3 Results

### 3.1 Generating interspecific hybrids through spore-to-spore mating

New interspecific hybrids were developed using spore-to-spore mating. Spores of domesticated *S. cerevisiae* beer strains BE031 and BE011, were crossed with wild *S. eubayanus* strains from Chile, DR1 and DR3, to generate *de novo* interspecific hybrids (Supplementary Figure S1). A species-specific PCR assay was used to confirm both parental genomes were present in the hybrids (Supplementary Figure S2). However, whereas the mating procedure was the same for each pair of parental strains, the hybridization rates differed (Table 1). Most notably, we failed to isolate any viable interspecific hybrids from parental strain BE031. Therefore, the rest of this manuscript will only include hybrids developed from BE011, DR1, and DR3.

**TABLE 1:** Overview of *de novo* interspecific hybrid yeasts generated.

	Result for hybridization with strain			
	**DR1**		**DR3**	
**Strain**	**No. of PCR-confirmed hybrids (designations)**	**Hybridization success rate (%)**	**No. of PCR-confirmed hybrids (designations)**	**Hybridization success rate (%)**
BE031	0	0 (0/576)	0	0 (0/432)
BE011	4 (H1–H4)	1.4 (4/288)	3 (H5–H7)	1.0 (3/288)

### 3.2 Selection of interspecific hybrids after being exposed in three different conditions

Next, we aimed to exploit the genome plasticity of newly developed hybrids to generate additional variability. It has been suggested that newly formed interspecific hybrids have unstable genomes, showing elevated rates of structural changes, including loss of large parts of their DNA as well as duplication events. These events gradually decline in frequency over time and/or mitotic cycles, resulting in more complex but stable hybrid genomes. Newly formed hybrids are therefore often grown for several generations to end up with variants that show more genetic and phenotypic stability, a procedure referred to as “genome stabilization.” We hypothesized that the short period of exceptional genome instability might offer an opportunity to rapidly generate further genetic and phenotypic diversity. We further hypothesized that carrying out the genome stabilization in specific environments might favor selection of those variants that are best adapted to the environment. To investigate if environmental conditions affect the result of the genome stabilization procedure and select for variants with different phenotypes, we subjected four parallel lineages of each hybrid to three different conditions (resulting in 7*4*3 = 84 stabilized lineages). The three conditions included 12°P wort at 10°C (LT); 12°P wort with addition of 8% (v/v) ethanol at 10°C (LT+Eth); and 12°P wort at 30°C (HT). Hybrids were transferred for 6 consecutive rounds, equivalent to 20–25 yeast generations.

**FIGURE 1:**
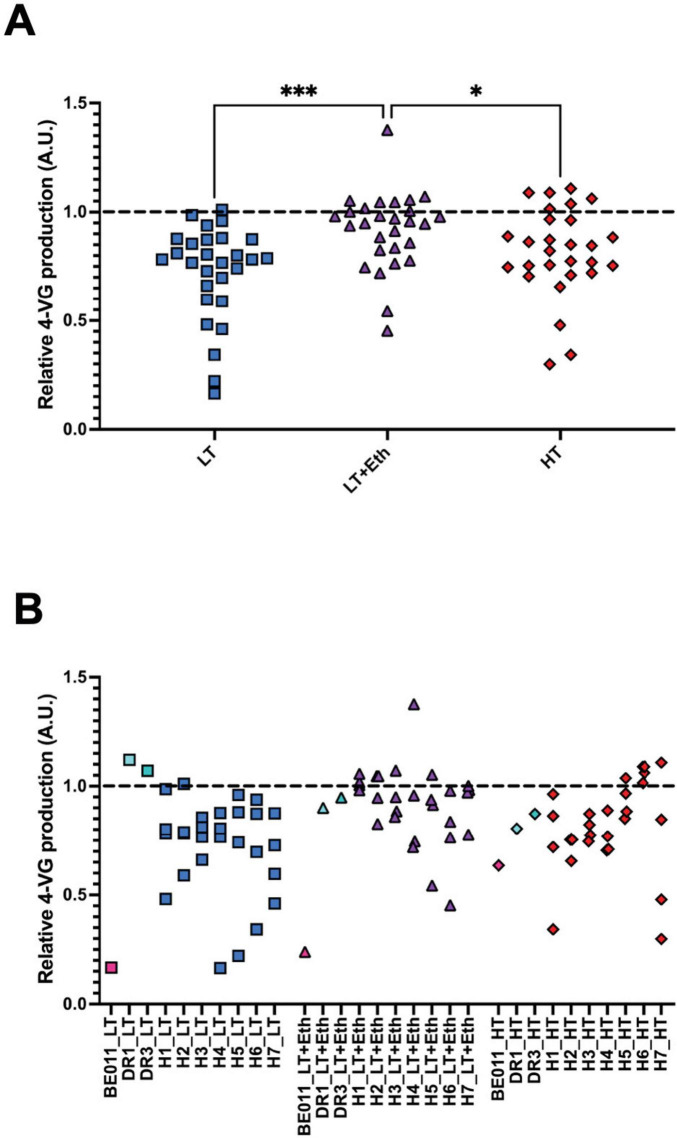
Screening of 4-vinyl guaiacol (4-VG) production. The conversion of ferulic acid to 4-VG by the stabilized lineages was measured by absorbance, visualized with lineages of all hybrids grouped **(A)**, or separated by hybrid **(B)**. In **(B)**, values for parental strains (stabilized in identical conditions as the hybrids) are also included. All the results were normalized to a scale of 0–1 based on performance of the 4-VG^–^ (set at 0) and 4-VG^+^ (set at 1) reference strains. Black dotted line represents the value of 4-VG^+^ reference strain). The asterisk indicate the level of significance (*p < 0.05; ***p < 0.001).

To select a subset of lineages to evaluate in mimicked lager beer fermentations, their capacity to metabolize ferulic acid, the precursor of the phenolic off-flavor 4-VG, after the stabilization procedure was assessed. We focus on this phenotype because 4-VG production has been reported to occur in all newly formed interspecific *S. pastorianus* lineages (Krogerus et al., 2015; Mertens et al., 2015), but is shown to have disappeared as a consequence of genome stabilization in Frohberg and Saaz yeasts (Gallone et al., 2019). On average, hybrids stabilized in LT+Eth showed significant higher levels of 4-VG production in comparison with the results obtained from LT (*p* < 0.001) and HT lineages (*p* < 0.05) (Figure 1A). While none of the lineages completely lost the ability to convert ferulic acid to 4-VG, some seem to show reduced activity (Figure 1B). Specifically, stabilized lineages of H7 showed the lowest average 4-VG production levels, with a high variability between the different stabilized lineages. This suggests that favorable changes might have occurred during genome stabilization in some, but not all, H7 lineages. Similarly, lineages of H5 showed high variability, with some lineages showing reduced 4-VG production levels. Therefore, both H5 and H7 were selected for further investigation.

### 3.3 Stabilized hybrids show variability in maltotriose utilization and flavor production

**FIGURE 2:**
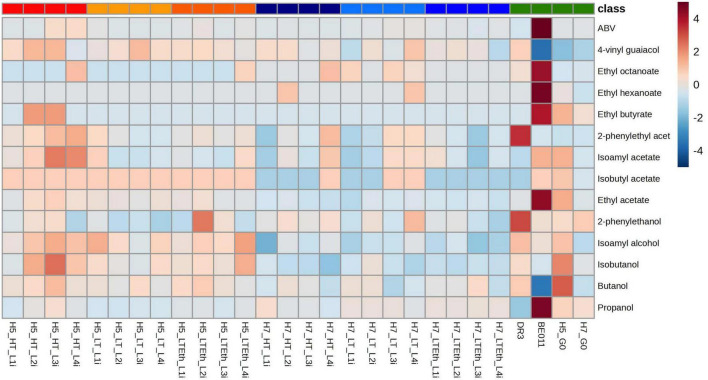
Heatmap representation of the metabolite production of stabilized isolates. Higher alcohols, esters, 4-VG and ABV produced after fermentation in lab-scale lager fermentations by stabilized isolates of interspecific hybrids H5 (red palette), H7 (blue palette) and the parental strains (BE011 and DR3) along with non-evolved hybrids (H5_G0 and H7_G0) (green) are given. Colors represent Z-scores (calculated over the rows).

To further investigate whether industrially relevant phenotypic differences emerged in stabilized lineages of H5 and H7, isolates of all these lineages (as well as the non-stabilized hybrids and parental strains) were subjected to lab-scale lager fermentation, and the concentrations of key fermentation metabolites were assessed (Table 2; Figure 2). Fermentation kinetics were monitored by daily tracking of the weight loss, which serves as a proxy for CO_2_ production and thus fermentation progress (Supplementary Figure S3). Between isolates, some variability in the CO_2_ loss occurred during the first 3 days of the fermentation, but total CO_2_ production at the end of the fermentation was similar for all isolates. Overall, alcohol by volume (% v/v) production of the stabilized isolates resemble those obtained with both *S. eubayanus* parental strains rather than the *S. cerevisiae* parents (Supplementary Figure S4). Interestingly, H5_HT_L3i and H5_HT_L4i showed slightly increased ABV production compared to the other stabilized isolates and its *S. eubayanus* parent. Analysis of the remaining sugar after fermentation showed that these strains are able to metabolize part of the maltotriose in the wort, while the non-stabilized hybrid (H5_G0) was unable to Supplementary Table S1).

**TABLE 2:** Overview of key compounds production from laboratory scale lager beer fermentation. CBS1513 and W34/70 are reference strains for Saaz- and Frohnerg-type lager yeasts, respectively.

	Propanol (mg L^–1^)	Butanol (mg L^–1^)	Isobutanol (mg L^–1^)	Isoamyl alcohol (mg L^–1^)	2-pheny lethanol (mg L^–1^)	Ethyl acetate (mg L^–1^)	Isobutyl acetate (mg L^–1^)	Isoamyl acetate (mg L^–1^)	2-phenylethyl acetate (mg L^–1^)	Ethyl butyrate (mg L^–1^)	Ethyl hexanoate (mg L^–1^)	Ethyl octanoate (mg L^–1^)	4-vinyl guaiacol (mg L^–1^)	Ethanol (% v v^–1^)
H5_LT_L1i	11.90	0.29	17.86	71.50	32.61	5.32	0.02	0.31	0.12	0.04	0.03	0.02	0.67	4.17
H5_LT_L2i	11.69	0.28	16.93	66.61	29.14	4.99	0.02	0.25	0.10	0.04	0.03	0.02	0.72	4.13
H5_LT_L3i	12.27	0.30	16.50	62.23	30.37	4.75	0.02	0.25	0.09	0.04	0.03	0.02	0.84	4.14
H5_LT_L4i	12.06	0.28	18.03	67.49	27.76	5.27	0.02	0.26	0.09	0.04	0.03	0.02	0.73	4.14
H5_LT+Eth_L1i	12.82	0.30	17.45	64.65	29.18	4.95	0.02	0.27	0.10	0.04	0.03	0.02	0.73	4.16
H5_LT+Eth_L2i	13.14	0.31	18.01	67.84	44.76	5.50	0.02	0.26	0.11	0.04	0.03	0.02	0.75	4.19
H5_LT+Eth_L3i	12.30	0.29	17.42	66.47	34.95	4.67	0.02	0.25	0.10	0.04	0.03	0.02	0.71	4.14
H5_LT+Eth_L4i	13.19	0.28	19.93	72.65	29.67	4.59	0.02	0.30	0.11	0.04	0.03	0.05	0.66	4.17
H5_HT_L1i	11.95	0.29	16.50	64.36	32.00	4.63	0.02	0.28	0.11	0.04	0.03	0.02	0.74	4.14
H5_HT_L2i	13.71	0.30	19.99	69.22	34.84	5.77	0.02	0.31	0.12	0.05	0.03	0.02	0.85	4.14
H5_HT_L3i	15.66	0.32	22.04	71.71	35.43	6.24	0.02	0.38	0.14	0.05	0.03	0.02	0.85	4.28
H5_HT_L4i	13.15	0.29	19.00	69.52	28.17	5.44	0.02	0.37	0.15	0.04	0.03	0.06	0.62	4.30
H7_LT_L1i	14.43	0.29	15.83	57.42	30.36	3.25	0.01	0.22	0.07	0.04	0.03	0.05	0.55	4.13
H7_LT_L2i	14.48	0.29	17.21	60.51	34.42	4.35	0.01	0.24	0.08	0.04	0.03	0.03	0.70	4.16
H7_LT_L3i	14.31	0.25	15.70	60.42	30.86	4.18	0.02	0.30	0.12	0.04	0.03	0.05	0.63	4.18
H7_LT_L4i	13.18	0.27	14.79	62.81	39.16	4.32	0.02	0.30	0.12	0.04	0.04	0.04	0.81	4.18
H7_LT+Eth_L1i	14.36	0.28	15.53	59.12	32.51	4.14	0.01	0.29	0.10	0.04	0.03	0.03	0.67	4.16
H7_LT+Eth_L2i	14.02	0.28	15.23	61.67	32.95	4.04	0.01	0.26	0.09	0.04	0.03	0.03	0.70	4.15
H7_LT+Eth_L3i	14.50	0.30	15.63	55.69	30.28	4.23	0.01	0.21	0.06	0.04	0.03	0.03	0.67	4.12
H7_LT+Eth_L4i	14.40	0.26	15.44	57.71	27.42	3.79	0.01	0.26	0.09	0.04	0.03	0.03	0.54	4.16
H7_HT_L1i	15.78	0.27	16.17	53.35	32.79	4.10	0.01	0.22	0.06	0.04	0.03	0.03	0.73	4.13
H7_HT_L2i	13.27	0.29	15.42	62.70	35.09	4.37	0.01	0.28	0.10	0.04	0.04	0.03	0.73	4.15
H7_HT_L3i	13.33	0.28	15.22	60.42	33.02	3.89	0.01	0.25	0.09	0.04	0.03	0.03	0.65	4.14
H7_HT_L4i	11.92	0.26	13.70	62.74	35.06	3.71	0.02	0.32	0.14	0.04	0.03	0.06	0.69	4.14
BE011	34.22	0.18	16.14	64.88	34.47	12.58	0.02	0.34	0.09	0.06	0.07	0.13	0.17	5.22
DR3	7.66	0.31	17.82	69.81	48.44	4.65	0.01	0.24	0.21	0.04	0.03	0.04	0.77	4.10
H5_G0	17.06	0.37	21.20	69.34	35.40	7.59	0.02	0.34	0.08	0.05	0.03	0.02	0.43	4.15
H7_G0	15.54	0.26	16.68	59.30	37.52	4.44	0.01	0.26	0.09	0.04	0.03	0.02	0.51	4.16
CBS1513	9.51	0.13	28.62	46.37	31.48	3.98	0.03	0.36	0.26	0.05	0.06	0.49	0.00	5.10
W34/70	11.37	0.21	19.82	54.19	34.25	4.28	0.02	0.34	0.13	0.05	0.05	0.21	0.00	4.96

H5_G0 and H7_G0 are the hybrids prior to genome stabilization. The values represent the mean of two technical replicates.

**FIGURE 3:**
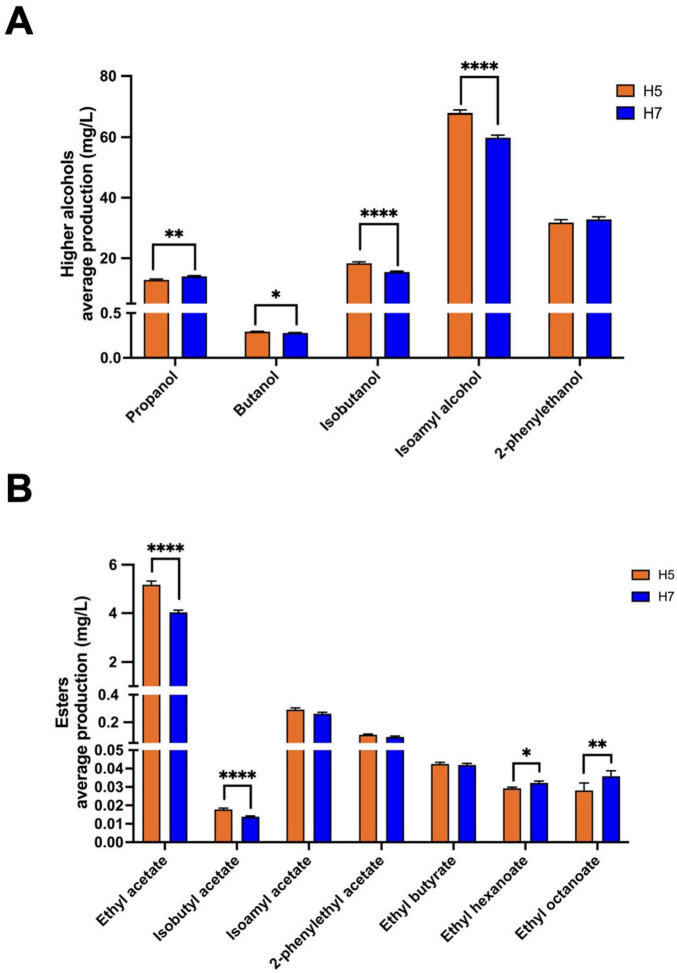
Production of higher alcohols and esters by stabilized isolates of H5 and H7. The average production of higher alcohols and their statistical differences observed between stabilized isolates of H5 (orange) and H7 (blue) is shown in **(A)**. Similarly, the average concentration of esters is shown in **(B)**. The asterisk indicate the level of significance (*p < 0.05; **p < 0.01; ****p < 0.0001).

**FIGURE 4:**
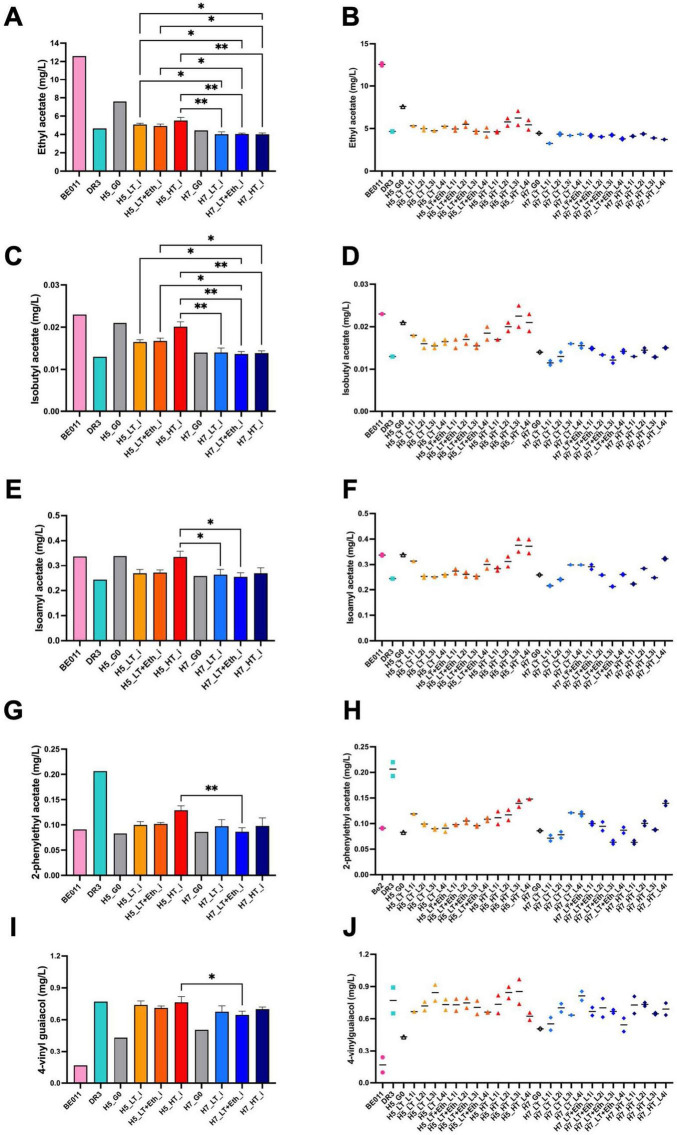
Production of acetate esters and 4-VG by stabilized isolates of H5 and H7. Production of ethyl acetate **(A,B)**, isobutyl acetate **(C,D)**, isoamyl acetate **(E,F)**, 2-phenylethyl acetate **(G,H)** and 4-vinyl guaiacol **(I,J)** of the stabilized isolates of H5 (orange palette) and H7 (blue palette) is depicted. The parental strains BE011 (pink) and DR3 (turquoise), and non-stabilized hybrids H5_G0 and H7_G0 (gray) are also included. In **(A,C,E,G,I)**, the mean value of stabilized isolates from all 4 parallel lineages is given. In **(B,D,F,H,J)**, the mean value of two technical replicates for each stabilized isolate is represented by a solid black line. The asterisk indicate the level of significance (*p < 0.05; **p < 0.01).

Aroma production differed significantly between stabilized isolates originating from either H5 or H7. While both groups are the result of the same parental combination (DR3 and BE011), this difference is likely explained by genetic differences in the segregants that led to both hybrids. For example, stabilized H5 isolates produced on average more isoamyl alcohol (67.85 vs. 59.82 mg/L, *p* < 0.0001), ethyl acetate (5.18 vs. 4.03 mg/L, *p* < 0.0001) and isobutyl acetate (0.018 vs. 0.014 mg/L, *p* < 0.0001), while stabilized H7 isolates produced on average higher concentrations of ethyl hexanoate (0.032 vs. 0.029 mg/L, *p* = 0.0269) and ethyl octanoate (0.036 vs. 0.028, *p* = 0.0035) (Figure 3). When comparing different genome stabilization strategies within the same hybrid, differences were in general more subtle. Most notably, isolates of H5 evolved at HT (H5_HT_i) seem to generally produce more esters (e.g., isoamyl acetate) compared to isolates stabilized in the two other conditions, but the trend is not statistically significant (Figure 4). All stabilized isolates maintained their capacity to produce 4-VG in concentrations above the 4-VG odor threshold described in pilsner beer (0.294 mg/L) (Vanbeneden et al., 2008; Figure 4J). However, significant differences were observed between 4-VG levels of H5 isolates stabilized in HT in comparison with H7 isolates after stabilization in LT+Eth (0.76 vs. 0.64 mg/L, *p* < 0.05).

**FIGURE 5:**
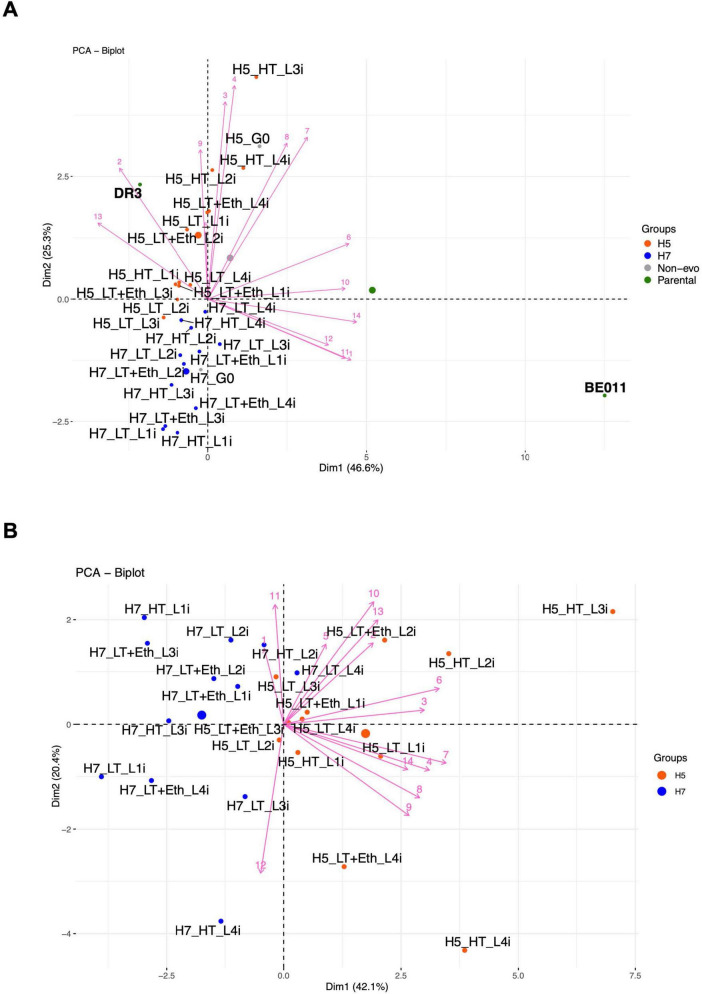
Two-dimensional (2-D) principal component analysis (PCA) separated by Dim1 = PC1 and Dim2 = PC2. Visualization of the metabolite production of stabilized H5 and H7 linages, non-stabilized hybrids and parental strains **(A)** and the metabolite production of only stabilized H5 and H7 lineages **(B)**. The component scores for the 13 volatile compounds are depicted as pink lines numbered as follows: 1, propanol; 2, butanol; 3, isobutanol; 4, isoamyl alcohol; 5, 2-phenylethanol; 6, ethyl acetate; 7, isobutyl acetate; 8, isoamyl acetate; 9, 2-phenylethyl acetate; 10, ethyl butyrate; 11, ethyl hexanoate; 12, ethyl octanoate; 13, 4-vinyl guaiacol; 14, alcohol by volume (ABV). Larger dots in the PCA plot correspond to the centroids of each group. 2-D PCA **(A,B)** explained 71.9% and 62.5% of the total variance, respectively.

Lastly, we performed principal component analysis (PCA) to visualize the overall diversity in aroma production profiles of the stabilized isolates, which revealed several additional interesting trends (Figure 5). Firstly, the aroma profile of all stabilized isolates mainly resembled that of the *S. eubayanus* parent, especially those originating from H5. Secondly, stabilized isolates of H5 and H7 generally cluster separately, mainly because of differences in higher alcohols and acetate esters production. Thirdly, after stabilization, the aroma profile of the hybrids deviates from that of the respective non-stabilized hybrid (H5_G0 and H7_G0) (Figure 5A), with some stabilized isolates from H5 (e.g., H5_LT_L2i and H5_LT_L3i) starting to resemble those of H7. After excluding both parental strains and the non-stabilized hybrid from the PCA to increase separation of the stabilized isolates, H5_HT_L3i and H5_HT_L4i seemed to produce the most distinct aroma profile (Figure 5B). Interestingly, these were also the hybrids showing the increased ethanol production (Table 2), further indicating significant changes occurred in the metabolism of these stabilized isolates.

## 4. Discussion

The generation of interspecific hybrids that combine and improve specific properties of the parental strains is a promising strategy to obtain superior industrial yeast variants (Gallone et al., 2019; Steensels et al., 2021). So far, several studies have demonstrated the potential of exploiting the available biodiversity in parental species. Careful selection of parent yeast strains that show desirable characteristics often allows generating hybrids that not only combine different properties of the parents, but even excel in some of the phenotypes (“best parent heterosis”) (Bernardes et al., 2017; Chen et al., 2023; Krogerus et al., 2017a; Meersman et al., 2015; Wauters et al., 2022). Interestingly, apart from exploiting the biodiversity in parental strains, the genomic plasticity of newly formed interspecific hybrids creates an additional opportunity to generate diversity among hybrids (Brickwedde et al., 2017; Gorter De Vries et al., 2019; Krogerus et al., 2018; Lancaster et al., 2019; Piotrowski et al., 2012). In this study, we generated 7 new interspecific hybrids through spore-to-spore mating of the domesticated *S. cerevisiae* strain BE011 (ale yeast from the “Beer 2” clade, Gallone et al., 2016) and two newly isolated wild *S. eubayanus* strains from Chile. Spore-to-spore mating with *S. cerevisiae* strain (BE031, from the “Beer 1” clade) proved unsuccessful. This suggests that stronger pre- or postzygotic mating barriers might be present for certain strains, which agrees with previous reported results (Krogerus et al., 2015; Mertens et al., 2015; Pérez-Través et al., 2012).

As a study case, we focused on generating hybrids with new aroma profiles. Apart from general fermentation and aroma characteristics, we specifically focused on the production of 4-VG, an undesirable aroma compound that is formed by all newly generated *S. cerevisiae* x *S. eubayanus* (lager) hybrids. Inspired by the fact that current industrial lager yeasts, which have been grown for hundreds of years since the hybridization event in the 16th century, have lost 4-VG production, we assessed whether specific genome stabilization procedures might cause changes in aroma profile in general, and 4-VG production in particular. We observed differences in aroma profile and 4-VG production between different stabilized isolates, with some showing lower 4-VG production. However, the stabilized isolates mostly showed an increase rather than decrease in 4-VG production compared to their non-stabilized ancestor (for example H5_HT_L4i, 44.4% increased). Further research is needed to elucidate if longer genome stabilization periods or different environments might better select for 4-VG negative strains. Nevertheless, the levels of 4-VG produced by the stabilized isolates are suitable for specific brewing beer styles such as Belgian specialty beers and wheat beers (Langos et al., 2013; Vanbeneden et al., 2008).

Interestingly, our results do suggest that certain stabilization regimes might allow selecting for improved fermentation characteristics. Specifically, while most newly formed interspecific hybrids did not show improvement in ethanol production (likely caused by the known inability of *S. eubayanus* to metabolize maltotriose), two stabilized isolates of H5 (H5_HT_L3i and H5_HT_L4i) produced higher ethanol concentrations, caused by improved maltotriose utilization. Emergence of maltotriose utilization in *S. eubayanus* has been observed previously, caused by the emergence of specific transporters of maltotriose by recombination (Baker and Hittinger, 2019; Brouwers et al., 2019). However, further studies are required to confirm if similar evolutionary events occurred in our hybrid lineages.

In addition, stabilized isolates showed differences in production of specific compounds compared to isolates stabilized in parallel, as well as compared to their parental strains. For example, H5_HT_L3i and H5_HT_L4i showed increased production of ethyl acetate, isobutyl acetate, isoamyl acetate and 2-phenylethyl acetate. This trend of increased acetate esters production after genome stabilization at increased temperature is intriguing. It has been suggested that fluidity of the plasma membrane (which is largely controlled by membrane lipid saturation) is affected by changes in temperature (Ferraz et al., 2021; Shinitzky and Henkart, 1979). At low temperature, the fluidity of the membrane is reduced and as a response, cells increase the degree of unsaturated fatty acids (Aguilera et al., 2007; Gibson et al., 2007), whereas the degree of unsaturation decreases at higher temperatures (Klose et al., 2012). Furthermore, it has been shown that high levels of unsaturated fatty acids repress the expression of *ATF1* and *ATF2* (alcohol acetyltransferases (AATase)) (Verstrepen et al., 2003a), which catalyze the synthesis of acetate esters in *Saccharomyces* (Verstrepen et al., 2003b). Therefore, it is tempting to speculate that the general trend of higher levels of the acetate esters in lineages evolved at high temperature (most notably H5_HT_L3i and H5_HT_L4i), can be explained by the lower levels of unsaturated fatty acids and subsequent increased gene expression of *ATF1* and *ATF2.*

In conclusion, by generating seven *de novo* interspecific yeast hybrids and stabilizing these in three different environmental conditions, we were able to demonstrate that diversity in the resulting hybrids is generated not only through the combination of the DNA of specific parental strains, but also during the mitotic cycles following the hybridization event. While we found that the overall effect of the different genome stabilization conditions that we used was rather subtle, we did find indications that genome stabilization at higher temperatures might increase acetate esters production, which could be a good strategy to produce strains for more fruity lager-type beers. Specifically, H5_HT_L3i and H5_HT_L4i might be the most interesting candidates due their 11.8 and 8.8% increase of isoamyl acetate production, and 3.1 and 3.6% increased ethanol production compared to their non-stabilized ancestor (H5_G0), while H5_HT_L4i also showed lower production of 4-VG in comparison to the other stabilized H5 isolates. However, whereas our study demonstrates the importance of the genome stabilization period for the development of the phenotypic characteristics of newly formed interspecific hybrids, several important questions merit further research. For example, incorporating current lager yeast strains could provide valuable insights into whether the adaptation speed is indeed accelerated in newly generated hybrids. Additionally, it would be interesting to investigate whether variation in hybrid ploidy influences adaptation, as ancestral lager strains were (likely) allotriploid or allotetraploid (Gallone et al., 2018). Moreover, following the genetic and phenotypic changes over a longer time, in more parallel lineages, and screening more isolates from each lineage, is needed to generate a better view on the extent, reproducibility and underlying molecular aspects of genome stabilization. Furthermore, genomic, transcriptomic and metabolomic investigation of the stabilized hybrids will be interesting to unravel the molecular mechanisms underlying the observed adaptations, which could provide insights in the general domestication process of interspecific hybrids, such as *S. pastorianus* lager yeast.

## Data Availability

The original contributions presented in the study are included in the article/Supplementary material, further inquiries can be directed to the corresponding authors.

## Footnotes

1
https://www.metaboanalyst.ca
