# Supplementary material for: Distinct genome stabilization procedures lead to phenotypic variability in newly generated interspecific yeast hybrids
Source: Front Microbiol. 2025 Jan 29;16:1472832. doi: 10.3389/fmicb.2025.1472832 (PMC11813950; doi:10.3389/fmicb.2025.1472832)
Supplement: Supplementary file 1 [file Table_1.docx]

| Table S1 | | | | | |
| --- | --- | --- | --- | --- | --- |
| Remaining sugars profile and ethanol production after fermentation of H5 isolates stabilized in HT. BE011 and DR3 are the *S. cerevisiae* and *S. eubayanus* parental strains, respectively. H5_G0 corresponds to H5 prior to genome stabilization. CBS1513 and W34/70 are reference strains of Saaz- and Frohberg-type lager yeasts, respectively. Values represent the average of two technical replicates, with standard deviations indicated. | | | | | |
|  | Glucose (g/L) | Sucrose (g/L) | Maltose (g/L) | Maltotriose (g/L) | Ethanol  (% v/v) |
| H5_HT_L1i | 0.239±0.007 | 0.113±0.005 | 0.062±0.000 | 14.523±0.0978 | 4.14±0.00 |
| H5_HT_L2i | 0.228±0.010 | 0.083±0.004 | 0.069±0.005 | 14.579±0.110 | 4.14±0.01 |
| H5_HT_L3i | 0.213±0.031 | 0.075±0.006 | 0.063±0.010 | 12.904±0.095 | 4.28±0.01 |
| H5_HT_L4i | 0.228±0.021 | 0.089±0.009 | 0.068±0.003 | 12.624±0.068 | 4.30±0.00 |
| BE011 | 0.317±0.008 | 0.152±0.009 | 0.037±0.005 | 1.532±0.231 | 5.22±0.02 |
| DR3 | 0.268±0.062 | 0.118±0.011 | 0.066±0.009 | 14.378±0.178 | 4.10±0.01 |
| H5_G0 | 0.258±0.033 | 0.072±0.015 | 0.059±0.010 | 15.002±0.501 | 4.15±0.01 |
| CBS1513 | 0.198±0.009 | 0.102±0.003 | 0.053±0.001 | 4.783±0.261 | 5.10±0.02 |
| W34/70 | 0.245±0.090 | 0.131±0.015 | 0.050±0.011 | 6.050±0.489 | 4.96±0.03 |
