# Supplementary material for: Distinct genome stabilization procedures lead to phenotypic variability in newly generated interspecific yeast hybrids
Source: Front Microbiol. 2025 Jan 29;16:1472832. doi: 10.3389/fmicb.2025.1472832 (PMC11813950; doi:10.3389/fmicb.2025.1472832)
Supplement: Supplementary file 2 [file Data_Sheet_1.pdf]

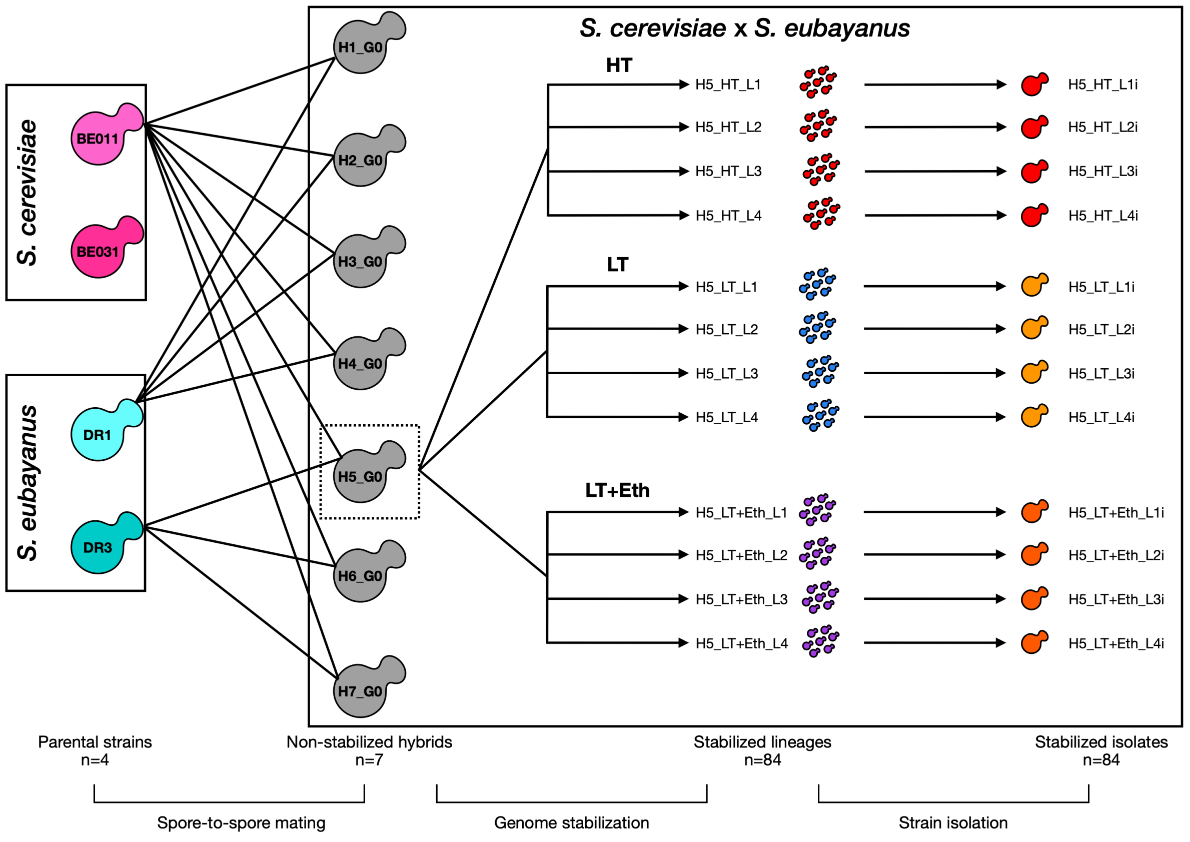

Supplementary Figure 1: Interspecific hybridization and genome stabilization procedure. First, two *S. cerevisiae strains* (BE011 and BE031) were crossed with two *S. eubayanus* strains (DR1 and DR3) through spore-to-spore mating to generate seven newly formed interspecific hybrids (H1-H7_G0). Then, four paralell lines from these hybrids were submitted to three different genome stabilization conditions: high temperature (HT), low temperature (LT) and low temperature with ethanol 8% v/v (LT+Eth). Later, the ferulic acid consumption from the stabilized hybrids pools was asssessed and stabilized hybrids isolates were selected for fermentation in lager-like brewing conditions.

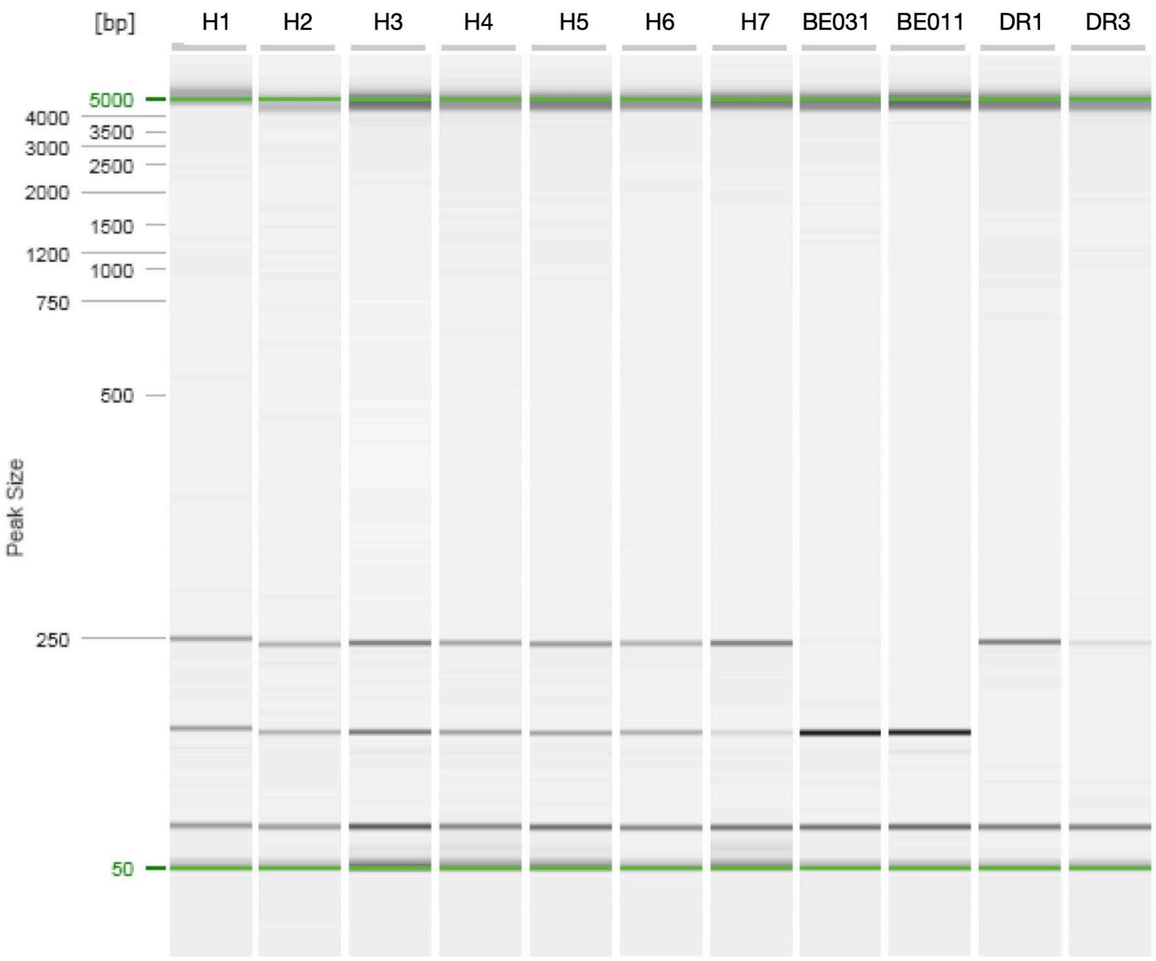

Supplementary Figure 2: Species-specific PCR. Species-specific PCR shows bands at 150 and 250 bp corresponding to *S. cerevisiae* and *S. eubayanus,* respectively. The seven new interspecific hybrids H1 to H7 showed the bands of both parental species.

A

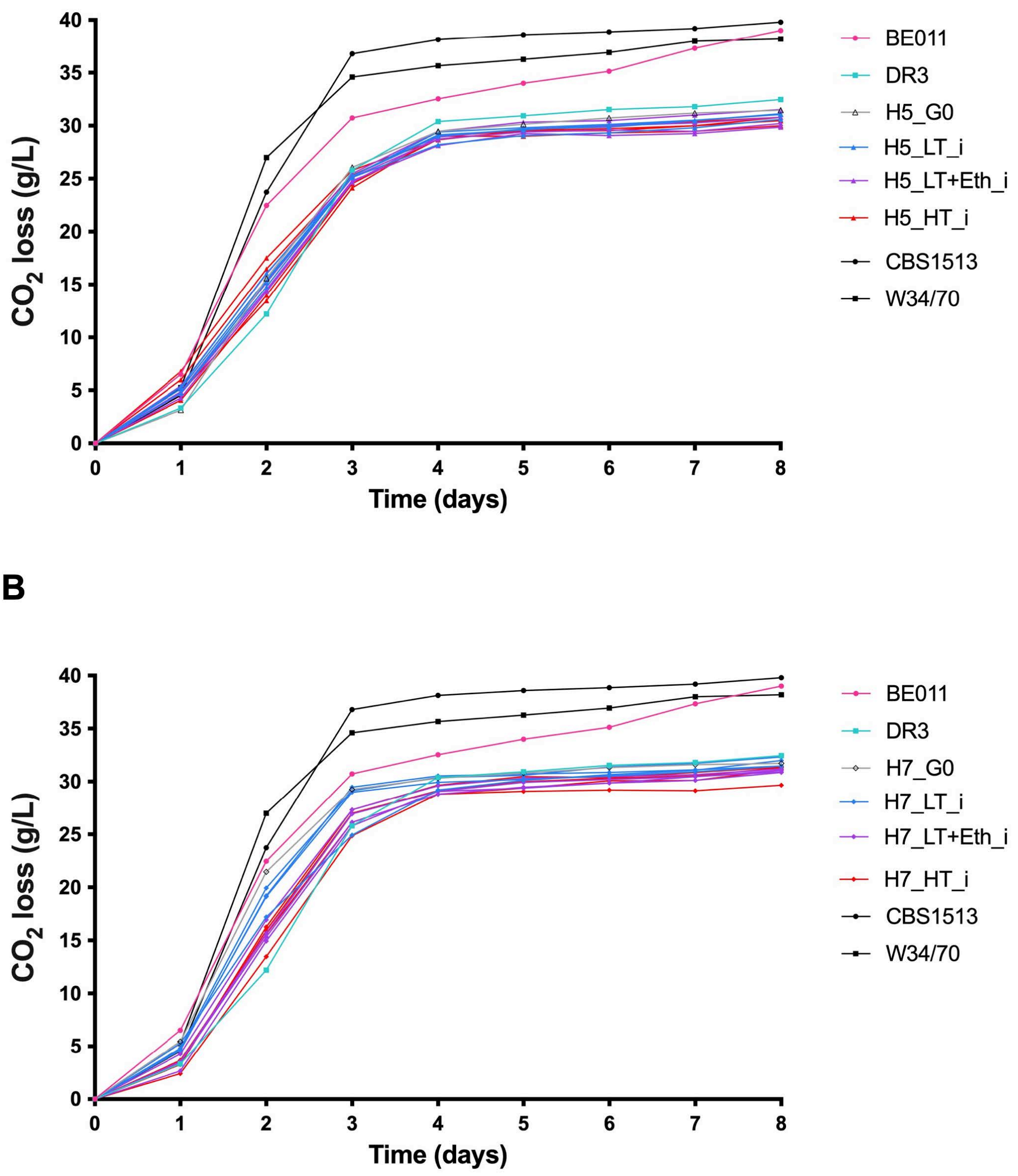

Supplementary Figure 3: Fermentation kinetics of H5 and H7. The weight loss (indicative of CO_2 production) of the individual lines of the interspecific hybrids H5 (colored triangles) (A) and H7 (colored diamonds) (B) after evolved in different environmental conditions: LT (blue), LT+Eth (purple) and HT (red) and non-devolved (uncolored); the parental strains *S. cerevisiae* BE011 (pink circle) and *S. eubayanus* DR3 (turquoise square); and the *S. pastorianus* reference strains Saaz (black circle) and Frohberg (black square) were daily monitored during 8 days.

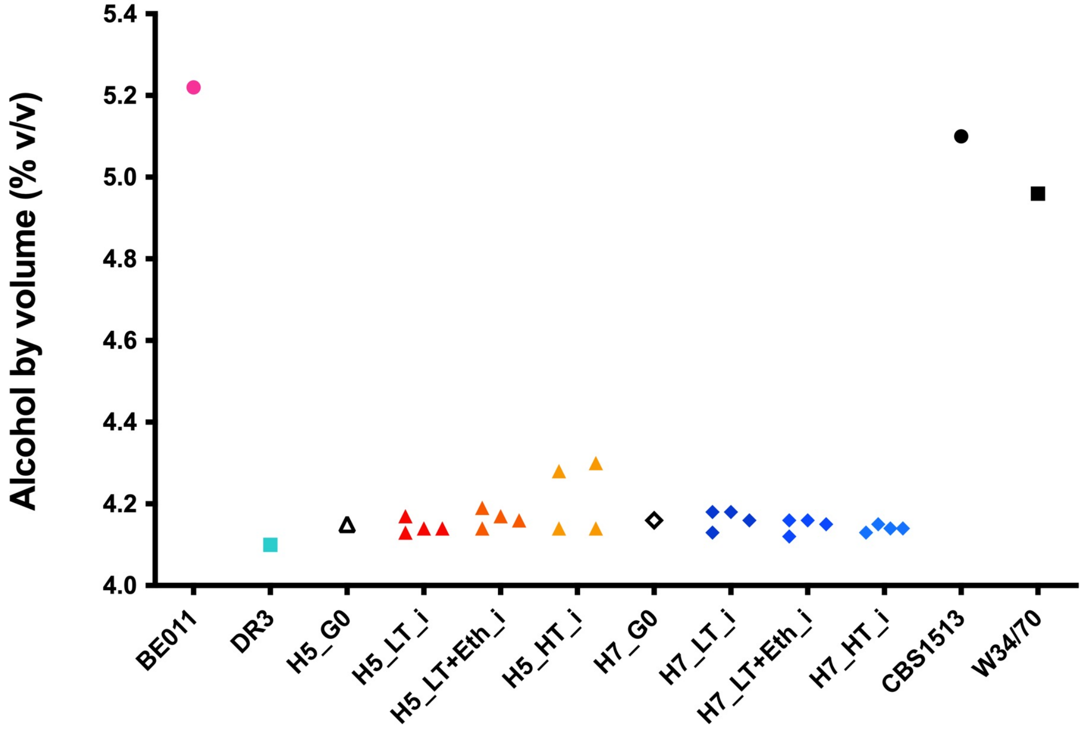

Supplementary Figure 4: Alcohol by volume (ABV) produced by interspecific hybrids. The figure shows the ABV produced during fermentation by the interspecific hybrids after being exposed to three different environmental conditions: H5 (orange pallete) and H7 (blue palette). The production of the non-volved hybrids (uncolored); the parental strains *S. cerevisiae* (pink) and *S. eubayanus* (turquoise); and the *S. pastorianus* reference strains Saaz (black circle) and Frohberg (black square) were also measured at the end of the fermentation.
